# Validity and reliability of the Japanese version of the Child and Adolescent Behavior Inventory: Parent version

**DOI:** 10.1002/pcn5.70066

**Published:** 2025-02-23

**Authors:** Ryusaku Hashimoto, Keitaro Sueda, Yui Tsuji, Yota Nakashima, Kazuyori Yagyu, Toshinobu Takeda

**Affiliations:** ^1^ Department of Communication Disorders School of Rehabilitation, Sciences, Health Sciences University of Hokkaido Hokkaido Japan; ^2^ Sapporo Child Physical & Mental Health Care Center Hokkaido Japan; ^3^ Sapporo Gakuin University Hokkaido Japan; ^4^ Department of Clinical Psychology Rakuwakai Otowa Hospital Otowa Japan; ^5^ Department of Child Development United Graduate School of Child Development, Osaka University Osaka Japan; ^6^ Department of Psychological Sciences Health Sciences University of Hokkaido Hokkaido Japan; ^7^ Department of Child and Adolescent Psychiatry Hokkaido University Graduate School of Medicine Hokkaido Japan; ^8^ Ryukoku University Kyoto Japan

**Keywords:** Child and Adolescent Behavior Inventory, cognitive disengagement syndrome, Japanese norm data, reliability, validity

## Abstract

**Aim:**

We developed a Japanese version of the Child and Adolescent Behavior Inventory (CABI) and evaluated its validity and reliability.

**Methods:**

A total of 2433 parents and 108 students in grades 4 and above participated in this study. First, we assessed the agreement between the parents' evaluations of internalizing disorders (anxiety and depression modules) and their children's self‐reports. The criterion‐related validity of attention deficit hyperactivity disorder (ADHD)‐inattention, ADHD‐hyperactivity/impulsivity, and oppositional defiant disorder (ODD) modules was also evaluated. Subsequently, we investigated whether cognitive disengagement syndrome (CDS) items differed from the ADHD items through factor analysis and examined the relationship between CDS and internalizing problems. Finally, we established normative data to adapt the CABI for use in the Japanese population.

**Results:**

Parents' observational evaluations of internalizing disorders showed moderate agreement with children's self‐reports. The ADHD‐inattention, ADHD‐hyperactivity/impulsivity, and ODD modules demonstrated moderate to high criterion‐related validity. Factor analysis revealed that the CDS module assesses symptoms distinct from ADHD symptoms. In the Japanese population, CDS exhibited a two‐factor structure (daydreaming and mental confusion), with developmental changes observed across age groups. The internal consistency and test–retest reliability of the CABI are moderate to high.

**Conclusions:**

The scores of each CABI module were found to be reliable and valid within the Japanese sample. Future clinical research should focus on the evaluation of CDS, which is often comorbid with ADHD.

## Graphical abstract

The questionnaires used in the survey and the plan for the number of distributions. The survey was conducted in two major time periods.

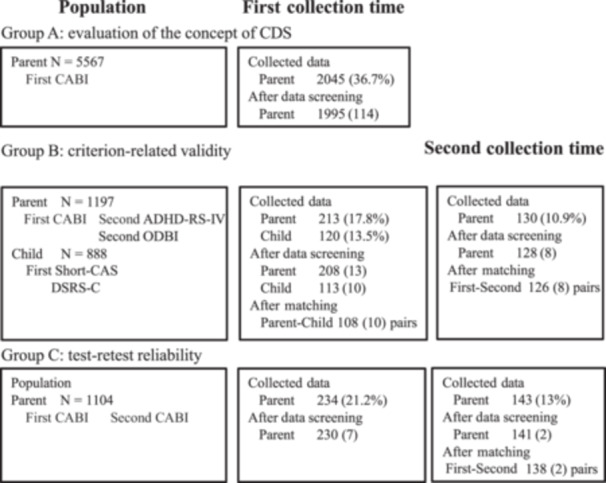

## INTRODUCTION

The Child and Adolescent Behavior Inventory (CABI) is used to evaluate several psychopathological symptoms.^1^ The CABI is divided into modules with a small number of questions. The module can be selected and used according to the user's purpose at no charge to researchers and clinicians. This could be a valuable tool for clinical screening and epidemiological studies.^2^ The CABI includes internalizing problems (anxiety and depression), externalizing problems (hyperactivity, impulsivity, and defiant attitude), and cognitive disengagement syndrome^3^ (CDS, formerly sluggish cognitive tempo). The CDS is characterized by daydreaming, mental confusion, and hypoactivity. The inattentive symptoms of attention‐deficit hyperactivity disorder (ADHD) and CDS are distinct.

The CABI has been translated and used in several countries (Spain,^4^ the United States,^2^ Iran,^5^ South Korea,^6^ Turkey^7^). While it can be used for international comparisons, a Japanese version has not been validated. In addition, internalizing problems are the subjective states of an individual, and it is not easy to assess them accurately, even for parents who live close to the individual. Therefore, it is necessary to determine the concordance between the parent's assessment (observation) and the individual's self‐assessment (subjective state). Furthermore, CDS is a newer concept than ADHD in Japan. It is reported that CDS is more closely associated with internalizing problems than with the inattention of ADHD. Therefore, it is necessary to investigate whether CDS symptoms can be considered independent and separable from ADHD symptoms in the Japanese population and to evaluate the relationships between CDS and internalizing problems.

This study aimed to develop a Japanese version of the CABI and examine the validity and reliability of each module. First, we evaluated the concordance between children's subjective evaluations of internalizing problems and those of their parents, and the criterion‐related validities of the traditionally used questionnaires. Second, we examined whether the CDS items were separated from ADHD items using factor analysis and whether the CDS related to internalizing problems. Finally, we provided norm data so that the CABI could be adapted for the Japanese population.

## METHODS

### Participants

**Figure 1:**
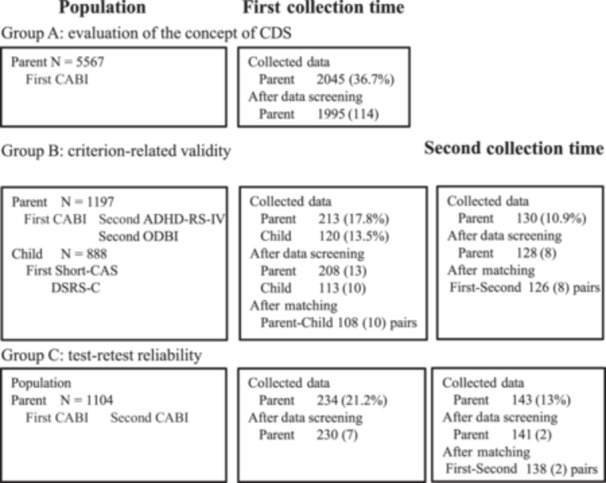
Distribution plan and collected data. Group A: CABI was distributed in December 2020 and collected in January 2021. Groups B and C: Two sets of questionnaires were distributed in July 2021 and collected twice: mid‐July to late July, and late August to early September 2021. Percentages in parentheses indicate response rates. Numbers represents participants diagnosed with any medical disorder or missing related data. CABI, Child and Adolescent Behavior Inventory; CDS, cognitive disengagement syndrome; ADHD‐RS‐IV, attention‐deficit/hyperactivity disorder‐rating scale‐IV; ODBI, oppositional defiant behavior inventory; Short‐CAS, short version of the Spence Children's Anxiety Scale; DSRS‐C, Birleson depression self‐rating scale for children.

This study was conducted in a city in Hokkaido, Japan. All public primary and junior high schools were divided into three groups. Group A was used to examine the concept of the CDS, and Groups B and C to evaluate validity and reliability (Figure 1). The study population consisted of parents or guardians of children (hereafter referred to as “parents”) in grades 1 through 9, and students in grade 4 and above. However, at the request of the City Board of Education, we did not include 9^th^‐grade students in Group A because they were preparing to take the high school entrance examination.

### Questionnaires

We utilized the following questionnaires and additionally asked parents if their children were medically diagnosed with neurological, developmental, or psychiatric disorders. Higher scores across all questionnaires indicate stronger traits of the respective conditions.

#### Parent

##### CABI

We used six modules of the CABI‐Parent version^1^: CDS, anxiety, depression, ADHD‐inattention (ADHD‐IN), ADHD‐hyperactivity/impulsivity (ADHD‐HI), and oppositional defiant disorder (ODD). A six‐point scale based on the quantified occurrence of each symptom was used to evaluate each module.

First, with Dr. Becker's permission, the draft of Japanese translation was prepared by clinicians with clinical experience in child psychiatry. Second, the draft was back translated by a bilingual English/Japanese translator. Finally, the draft was reviewed by Dr. Becker and the final version was created.

##### ADHD‐RS‐IV

We used the Japanese version of the ADHD‐Rating Scale‐IV.^8^ It consists of 18 items, nine for ADHD‐IN and nine for ADHD‐HI, rated on a four‐point scale. The total scores for ADHD‐IN and ADHD‐HI range from 0 to 27.

##### ODBI

The Oppositional Defiant Behavior Inventory (ODBI) is a parent rating scale for oppositional behavior.^9^ ODBI has 18 items rated on a four‐point scale, with a total score ranging from 0 to 54.

#### Child

##### Short CAS

The Japanese version of the short version of the Spence Children's Anxiety Scale (Short CAS) consists of eight items rated on a four‐point scale ranging from “never” to “always.”^10^ The total score ranges from 0 to 24.

##### DSRS‐C

We used the short form of the Birleson Depression Self‐Rating Scale for Children (DSRS‐C), which was developed and verified based on item response theory.^11^ DSRS‐C has nine items rated on a three‐point scale ranging from “always” to “never.” The total score ranges from 0 to 18.

### Data analysis

#### Data quality control

We excluded responses with missing demographic data (sex, age, and grade) and responses with two or more missing values in CDS, ADHD‐IN, ADHD‐HI, ODD modules, ADHD‐RS‐IV, and OBDI. Additionally, we excluded responses with missing values in the Anxiety and Depression modules, Short CAS, and DSRS‐C. Finally, we analyzed the missing values and imputed them using the ME method with SPSS Missing Values software (IBM).

#### Validation

To clarify the agreement between parent's and child's ratings on anxiety and depression, Pearson's correlation coefficients of the Anxiety and Depression modules were calculated using the child‐rated Short CAS and DSRS‐C. Additionally, correlation coefficients for the ADHD‐IN, ADHD‐HI, and ODD modules were computed to assess criterion‐related validity against traditionally used questionnaires.

#### Concept of CDS

We employed exploratory factor analysis on data from Group A to determine whether CDS items could be distinguished as an independent factor from ADHD‐related items. Subsequently, we calculated Pearson's correlation coefficients to determine the possible relationship of CDS score and internalizing problems.

#### Reliability

For internal consistency reliability, we calculated Cronbach's *α* for each CABI module based on Group A. For test–retest reliability, we calculated intraclass correlation coefficient (ICC) values for each CABI module based on the matched data of Group C.

#### Sex and grade differences in CDS

First, if any medical condition was described in responses, the medical doctors reviewed and excluded it from subsequent data analysis. The data missing the medical condition were also excluded. Second, we compared sampling periods (Group A vs. Groups B and C) to confirm no differences existed during the COVID‐19 pandemic. If no primary effect was observed, the three groups were combined. Anticipating a small sample size for the 9th grade, we combined the 9th and 8th grades. We performed a two‐way analysis of variance (ANOVA) with sex and grade level as factors. We present the normative data based on the combined data.

Additionally, to estimate the comorbidity of CDS and ADHD symptoms, we classified all samples into no symptoms, single symptoms, or CDS with any ADHD symptoms categories, using the 95th percentile cutoff values of each sex and grade. Children who scored equal to or above the cutoff value were identified as exhibiting characteristics of CDS, ADHD‐IN, or ADHD‐HI. For instance, if a child's scores for both CDS and ADHD‐IN surpassed the cutoff values, the child was categorized as “CDS with ADHD‐IN,” indicating that their ADHD‐HI score remained below the cutoff threshold.

We used SPSS Statistics 25, Missing Values, and Amos 29 for all statistical analyses, with the significance set at P < 0.05. If multiple comparisons were needed in a post hoc test, we used the Bonferroni procedure with significance.

## RESULTS

### Data quality control (Figure 1 and Table 1)

Based on data quality control, we excluded 50 responses (2.4%) from Group A, leaving 1995 parent responses for analysis. In Group B, we excluded five parent responses (2.3%) and seven child responses (5.8%) during the first collection period, as well as two parent responses (1.5%) during the second collection period. We matched 108 parent–child pairs and 126 first–second collection time pairs for analysis. In Group C, we excluded four responses (1.7%) and two responses (1.5%) during each collection period, and matched 138 pairs for analysis.

**Table 1:** Number of samples in each group.

Group responder	Sex	Primary school												Junior high school						Total	
		Grade 1		2		3		4		5		6		7		8		9			
Group A Parent	Boy	163	(12)	124	(7)	128	(11)	124	(7)	90	(6)	115	(5)	94	(7)	101	(9)	‐		939	(64)
	Girl	147	(2)	133	(3)	140	(11)	150	(7)	133	(4)	128	(9)	110	(9)	115	(5)	‐		1056	(50)
	Total	310	(14)	257	(10)	268	(22)	274	(14)	223	(10)	243	(14)	204	(16)	216	(14)	‐		1995	(114)
Group B Parent	Boy	12	(1)	12		23	(1)	8		10	(3)	8	(1)	9	(1)	17	(3)	5	(1)	104	(11)
First collection time	Girl	12		12		14		12		10		10	(1)	8		19		7	(1)	104	(2)
	Total	24	(1)	24		37	(1)	20		20	(3)	18	(2)	17	(1)	36	(3)	12	(2)	208	(13)
Second collection time	Boy	8		9		13	(1)	5		4	(3)	3		4		10	(1)	3	(1)	59	(6)
	Girl	9		12		8		4		8		6	(1)	7		9		6	(1)	69	(2)
	Total	17		21		21	(1)	9		12	(3)	9	(1)	11		19	(1)	9	(2)	128	(8)
Group B Child	Boy	‐		‐		‐		7		8	(3)	5	(1)	7		17	(3)	6	(1)	50	(8)
First collection time	Girl	‐	‐	‐		‐		8		10		10	(1)	9		17		9	(1)	63	(2)
	Total	‐		‐		‐		15		18	(3)	15	(2)	16		34	(3)	15	(2)	113	(10)
Group C Parent	Boy	23		16		15		16		19		13	(3)	7	(1)	8	(1)	5		122	(5)
First collection time	Girl	14		10		16	(2)	19		15		12		7		9		6		108	(2)
	Total	37		26		31	(2)	35		34		25	(3)	14	(1)	17	(1)	11		230	(7)
Second collection time	Boy	15		8		14		11		10		8		3	(1)	5	(1)	1		75	(2)
	Girl	10		4		10		12		13		8		2		5		2		66	
	Total	25		12		24		23		23		16		5	(1)	10	(1)	3		141	(2)

*Note*: Numbers in parentheses indicate participants were who diagnosed with any medical disorder or [‐] means have no data about it.

### Validation (Table 2)

The parent‐rated Anxiety and Depression modules demonstrated a moderate correlation with their children's scores on the short‐CAS and DSRS‐C. The ADHD‐IN, ADHD‐HI, and ODD modules were strongly correlated with the ADHD‐RS‐IV and ODBI rated by the same parents.

**Table 2:** Correlation coefficients: CABI modules versus questionnaires.

CABI module	Paired questionnaire	Correlation coefficient
*Parent with child correlations (N = 108)*		
Anxiety	Short‐CAS	0.49
Depression	DSRS‐C	0.52
*Parent with parent correlations (N = 126)*		
ADHD‐IN	ADHR‐RS‐IV (inattention items)	0.81
ADHD‐HI	ADHD‐RS‐IV (hyperactivity/impulsive items)	0.71
ODD	ODBI	0.75

Abbreviations: ADHD‐HI, attention‐deficit/hyperactivity disorder‐hyperactivity/impulsivity; ADHD‐IN, attention‐deficit/hyperactivity disorder‐inattention; ADHD‐RS‐IV, attention‐deficit/hyperactivity disorder‐rating scale‐IV; DSRS‐C, Birleson depression self‐rating scale for children; ODBI, oppositional defiant behavior inventory; ODD, oppositional defiant disorder; Short‐CAS, short version of Spence children's anxiety scale.

*Note*: All P‐values were <0.001.

### Concept of CDS (Tables 3 and 4)

We conducted an exploratory factor analysis (EFA) and determined the number of factors to be four using parallel analysis. The maximum likelihood method was used for extraction, with oblique rotation (promax rotation) applied, as the factors were assumed to be correlated. The EFA showed distinct factors for ADHD‐HI and ADHD‐IN items (Table 3). All ADHD factor loadings exceeded 0.5, whereas the factor loadings for other factors were below 0.3. Two factors were extracted for the CDS items. In Factor 3, two CDS items had factor loadings below 0.5 (CDS 1: 0.47, CDS 7: 0.49); the rest exceeded 0.5. Factor loadings for other factors were below 0.3. In Factor 4, one item had a factor loading below 0.5 (CDS 6: 0.49); the rest were above 0.5. Additionally, one item had a factor loading 0.31 for Factor 2 (CDS 14). Factor correlations ranged from 0.26 to 0.70. All Cronbach's *α* coefficients were above 0.86. Based on item content, Factors 3 and 4 were named as “daydreaming” and “mental confusion,” respectively.

**Table 3:** Result of the exploratory factor analysis.

CABI module	Factor loading			
Item number	Factor 1	Factor 2	Factor 3	Factor 4
ADHD‐HI 4	**0.80**	−0.12	−0.03	0.08
ADHD‐HI 5	**0.78**	0.04	−0.06	0.02
ADHD‐HI 7	**0.77**	−0.02	0.02	0.03
ADHD‐HI 6	**0.76**	−0.07	0.05	0.01
ADHD‐HI 9	**0.73**	0.01	0.01	0.03
ADHD‐HI 3	**0.70**	0.07	−0.05	−0.06
ADHD‐HI 8	**0.63**	0.04	−0.01	0.01
ADHD‐HI 2	**0.53**	0.23	0.02	−0.11
ADHD‐HI 1	**0.53**	0.18	0.05	0.03
ADHD‐IN 2	0.00	**0.91**	−0.03	−0.05
ADHD‐IN 6	−0.01	**0.90**	−0.02	−0.09
ADHD‐IN 4	0.05	**0.88**	0.01	−0.13
ADHD‐IN 8	0.08	**0.84**	0.03	−0.05
ADHD‐IN 5	0.01	**0.77**	−0.07	0.17
ADHD‐IN 7	0.06	**0.70**	0.00	0.02
ADHD‐IN 9	0.02	**0.67**	0.00	0.18
ADHD‐IN 1	0.07	**0.65**	0.01	0.14
ADHD‐IN 3	0.13	**0.55**	0.15	0.05
CDS 08	0.01	−0.11	**0.83**	−0.06
CDS 03	0.03	0.00	**0.78**	−0.11
CDS 02	0.01	−0.02	**0.74**	0.03
CDS 05	0.10	−0.05	**0.73**	−0.08
CDS 12	−0.01	−0.01	**0.71**	0.16
CDS 09	0.00	−0.02	**0.53**	0.05
CDS 04	−0.02	0.02	**0.51**	−0.05
CDS 07	−0.13	0.13	**0.49**	0.06
CDS 01	−0.07	0.16	**0.47**	0.07
CDS 13	0.06	−0.07	−0.10	**0.96**
CDS 11	0.08	−0.12	−0.03	**0.90**
CDS 15	−0.02	0.11	−0.01	**0.67**
CDS 10	0.00	0.01	0.12	**0.63**
CDS 14	−0.17	**0.31**	0.08	**0.58**
CDS 06	−0.01	0.08	0.24	**0.49**
Factor correlations				
Factor 1	‐	0.62	0.26	0.40
Factor 2		‐	0.55	0.70
Factor 3			‐	0.63

*Note*: Bold values represent factor loadings greater than 0.3.

Abbreviations: ADHD‐HI, attention‐deficit/hyperactivity disorder‐hyperactivity/impulsivity; ADHD‐IN, attention‐deficit/hyperactivity disorder‐inattention; CABI, Child and Adolescent Behavior Inventory; CDS, cognitive disengagement syndrome.

The scores of CDS, ADHD‐IN, and ADHD‐HI modules correlated with the Anxiety, Depression, and ODD modules. After controlling for ADHD‐IN as a covariate, CDS was found to be correlated with the Anxiety and Depression modules, but not with ODD, whereas ADHD‐HI was only correlated with ODD module (Table 4).

**Table 4:** Correlation among CDS, ADHD, and other modules.

Module	Anxiety	Depression	ODD
CDS	0.49	0.49	0.37
ADHD‐IN	0.40	0.41	0.50
ADHD‐HI	0.27	0.26	0.51
*After controlling for ADHD‐IN as a covariate*			
CDS	0.33	0.32	0.06 ns
ADHD‐HI	0.03 ns	0.00 ns	0.28

*Note*: To avoid a multiple comparison, the P‐value was set at 0.01.

Abbreviations: ADHD‐IN, attention‐deficit/hyperactivity disorder‐inattention; ADHD‐HI, attention‐deficit/hyperactivity disorder‐hyperactivity/impulsivity; CDS, cognitive disengagement syndrome; ODD, oppositional defiant disorder; ns, nonsignificant.

### Reliability (Table 5)

The Cronbach's *α* values for each CABI module, daydreaming, and mental confusion exceeded 0.80. The mean interval between test and retest was 48 days (standard deviation 8.9 days). Except for the Depression module, the ICCs for most modules, daydreaming, and mental confusion were ∼0.8.

**Table 5:** Internal consistency and reproducibility for CABI modules.

CABI module	Cronbach's *α*	ICC	95% CI
CDS total	0.91	0.81	0.74–0.86
Daydreaming	0.86	0.79	0.72–0.85
Mental confusion	0.90	0.79	0.72–0.84
Anxiety	0.80	0.76	0.68–0.82
Depression	0.86	0.57	0.45–0.67
ADHD‐IN	0.95	0.88	0.84–0.92
ADHD‐HI	0.90	0.81	0.74–0.86
ODD	0.92	0.80	0.73–0.85

Abbreviations: ADHD‐IN, attention‐deficit/hyperactivity disorder‐inattention; ADHD‐HI, attention‐deficit/hyperactivity disorder‐hyperactivity/impulsivity; CABI, Child and Adolescent Behavior Inventory; CDS, cognitive disengagement syndrome; CI, confidence interval; ICC, intraclass correlation coefficient; ODD, oppositional defiant disorder.

### Sex and grade differences in CDS (Tables 6 and 7)

To clarify typical developmental changes, we excluded 134 responses (5.5%) diagnosed with any medical disorder or missing related data (Group A = 1881, B = 195, C = 223). To assess differences based on the sampling period, we performed a three‐way ANOVA (period, sex, and grade). A three‐way ANOVA revealed no main effect of Period (all P > 0.1) and no interactions between Period and Grade or Sex in the CABI modules (all P > 0.05). Therefore, we combined three groups for the subsequent analysis (*N* = 2299).

**Table 6:** Mean scores for each sex and grade level.

	Boy					Girl						Boy					Girl				
			Percentile ranking					Percentile ranking						Percentile ranking					Percentile ranking		
Grade	Mean	SE	85	90	95	Mean	SE	85	90	95	Grade	Mean	SE	85	90	95	Mean	SE	85	90	95
*CDS total*											*Depression*										
1st	8.79	0.73	19.0	23.0	29.7	8.71	0.76	18.0	22.8	34.0	1st	0.88	0.18	2.0	3.0	5.0	0.85	0.19	2.0	3.0	4.0
2nd	8.91	0.82	18.1	25.0	29.7	8.14	0.80	17.0	20.0	31.4	2nd	1.22	0.21	3.0	4.4	8.0	1.03	0.20	2.0	4.0	5.0
3rd	8.48	0.80	19.9	23.0	31.2	7.79	0.79	16.0	18.2	24.1	3rd	0.87	0.20	1.9	2.0	4.3	0.94	0.20	2.0	3.0	5.1
4th	6.85	0.84	14.0	18.8	24.9	7.86	0.75	15.0	17.5	23.3	4th	1.09	0.21	3.0	3.8	6.0	0.95	0.19	2.0	3.0	4.3
5th	9.93	0.95	20.0	25.9	31.0	7.87	0.80	17.0	21.0	24.0	5th	1.01	0.24	2.0	4.0	6.0	0.97	0.20	2.0	3.0	5.3
6th	8.88	0.88	18.8	24.0	30.0	9.41	0.84	18.9	23.8	35.9	6th	1.19	0.22	3.0	4.0	6.0	1.41	0.21	3.0	5.0	6.9
7th	9.50	0.98	21.6	29.0	38.9	11.19	0.92	20.9	26.2	32.5	7th	1.17	0.25	3.0	4.7	7.7	1.99	0.23	5.0	6.0	9.3
8–9th	7.85	0.90	17.6	22.0	29.9	9.80	0.81	22.0	27.0	35.0	8–9th	1.32	0.23	3.0	4.0	9.0	2.22	0.20	5.0	7.0	15.0
*Daydreaming*											*ADHD‐IN*										
1st	4.14	0.44	9.0	12.0	15.4	4.67	0.45	9.4	13.0	17.4	1st	11.98	0.69	24.1	29.0	34.0	9.77	0.72	18.0	22.6	31.0
2nd	4.08	0.49	9.0	11.4	15.7	4.24	0.48	9.0	12.0	17.0	2nd	12.74	0.78	25.1	30.4	35.7	9.48	0.76	19.1	22.7	28.4
3rd	4.02	0.48	8.0	12.6	18.0	4.22	0.47	9.0	11.2	16.1	3rd	11.26	0.76	24.0	27.0	36.6	8.94	0.75	19.0	24.4	32.0
4th	3.35	0.50	7.0	9.0	13.9	4.04	0.45	9.0	10.0	13.0	4th	9.33	0.79	17.0	24.0	31.9	7.61	0.71	16.8	20.0	27.0
5th	5.05	0.57	9.3	13.9	17.5	4.55	0.48	10.0	12.5	14.5	5th	10.51	0.90	23.4	27.0	32.9	5.80	0.76	13.0	17.0	21.0
6th	5.54	0.53	13.0	15.2	21.6	5.73	0.50	11.0	15.8	19.0	6th	9.08	0.83	19.0	25.0	29.0	6.34	0.79	13.0	17.9	24.9
7th	5.50	0.59	12.6	16.7	21.0	6.91	0.55	14.0	16.0	21.5	7th	8.09	0.93	17.6	22.7	32.9	7.94	0.87	16.0	19.6	33.0
8–9th	5.17	0.54	11.1	16.0	19.9	6.43	0.49	12.0	20.0	23.0	8–9th	6.73	0.85	16.0	18.0	24.4	5.65	0.77	12.4	16.9	21.9
*Mental confusion*											*ADHD‐HI*										
1st	4.65	0.37	10.0	11.0	16.0	4.04	0.39	9.0	11.8	16.8	1st	6.91	0.42	13.0	18.0	23.7	4.04	0.44	8.2	11.8	15.4
2nd	4.83	0.42	11.0	13.0	16.4	3.89	0.41	9.0	11.0	16.0	2nd	7.26	0.47	17.0	20.0	27.1	3.95	0.46	9.0	15.7	19.4
3rd	4.46	0.41	10.0	12.6	18.0	3.57	0.40	7.0	9.0	13.0	3rd	5.48	0.46	11.0	15.0	22.3	2.71	0.46	6.0	8.2	12.3
4th	3.50	0.42	8.0	9.8	12.9	3.82	0.38	8.0	10.0	14.0	4th	4.20	0.48	9.0	12.8	17.0	2.70	0.43	6.0	7.5	14.0
5th	4.88	0.48	11.0	13.0	17.8	3.32	0.41	7.0	9.0	12.0	5th	4.35	0.54	9.0	14.7	17.9	1.64	0.46	4.0	5.0	9.0
6th	3.35	0.45	8.0	10.0	11.6	3.68	0.43	8.8	10.0	16.9	6th	3.69	0.51	8.8	13.0	16.0	1.31	0.48	3.0	5.0	8.0
7th	4.00	0.50	10.6	11.7	17.9	4.28	0.47	9.0	12.3	17.2	7th	2.53	0.57	7.0	8.0	11.0	2.14	0.53	3.0	5.0	14.0
8th	2.68	0.46	6.6	9.0	12.9	3.37	0.41	8.0	10.0	15.5	8–9th	1.30	0.52	2.0	3.7	8.0	1.17	0.47	3.0	4.0	7.0
*Anxiety*											*ODD*										
1st	2.66	0.28	5.0	6.0	9.7	3.19	0.29	6.2	8.0	13.0	1st	6.26	0.49	13.1	15.0	23.4	4.95	0.51	11.0	13.0	20.0
2nd	3.02	0.32	7.0	10.0	12.0	3.72	0.31	7.0	9.0	13.4	2nd	5.44	0.56	10.1	16.0	20.0	4.62	0.54	10.0	13.7	19.0
3rd	2.46	0.31	6.0	7.0	11.0	3.14	0.30	7.0	9.2	13.0	3rd	4.70	0.54	9.0	11.6	19.9	4.91	0.54	9.3	14.2	22.0
4th	2.73	0.32	6.0	7.0	9.9	3.03	0.29	7.0	8.0	10.3	4th	5.73	0.57	13.0	17.6	21.9	4.57	0.51	9.0	13.0	19.3
5th	2.47	0.36	5.0	7.9	11.0	2.53	0.31	5.0	6.0	9.0	5th	5.04	0.64	10.4	14.0	21.9	4.25	0.54	9.0	11.0	16.3
6th	2.56	0.34	6.0	7.0	10.0	3.05	0.32	6.8	8.0	10.0	6th	4.58	0.60	9.0	10.2	16.6	4.50	0.57	9.0	11.0	19.9
7th	2.58	0.38	5.6	8.7	11.0	3.02	0.35	6.0	8.0	12.2	7th	4.44	0.67	9.6	13.7	16.0	5.74	0.62	13.0	14.6	22.0
8–9th	1.80	0.34	4.0	5.0	8.0	3.12	0.31	6.3	9.0	12.5	8–9th	3.93	0.61	9.6	12.0	15.0	4.39	0.55	8.0	12.8	21.5

Abbreviations: ADHD‐IN, attention‐deficit/hyperactivity disorder‐inattention; ADHD‐HI, attention‐deficit/hyperactivity disorder‐hyperactivity/impulsivity; CDS, cognitive disengagement syndrome; ODD, oppositional defiant disorder; SE, standard error.

**Table 7:** Comorbidity of CDS and ADHD symptoms.

CDS comorbidity				ADHD only		
None	ADHD‐IN	ADHD‐HI	ADHD‐CON	IN	HI	CON
61	41	9	28	50	78	28

*Note*: The number of all samples was 2433.

Abbreviations: ADHD, attention‐deficit/hyperactivity disorder; CDS, cognitive disengagement syndrome; CON, combined IN and HI; HI, hyperactivity/impulsivity; IN, inattention.

**Figure 2:**
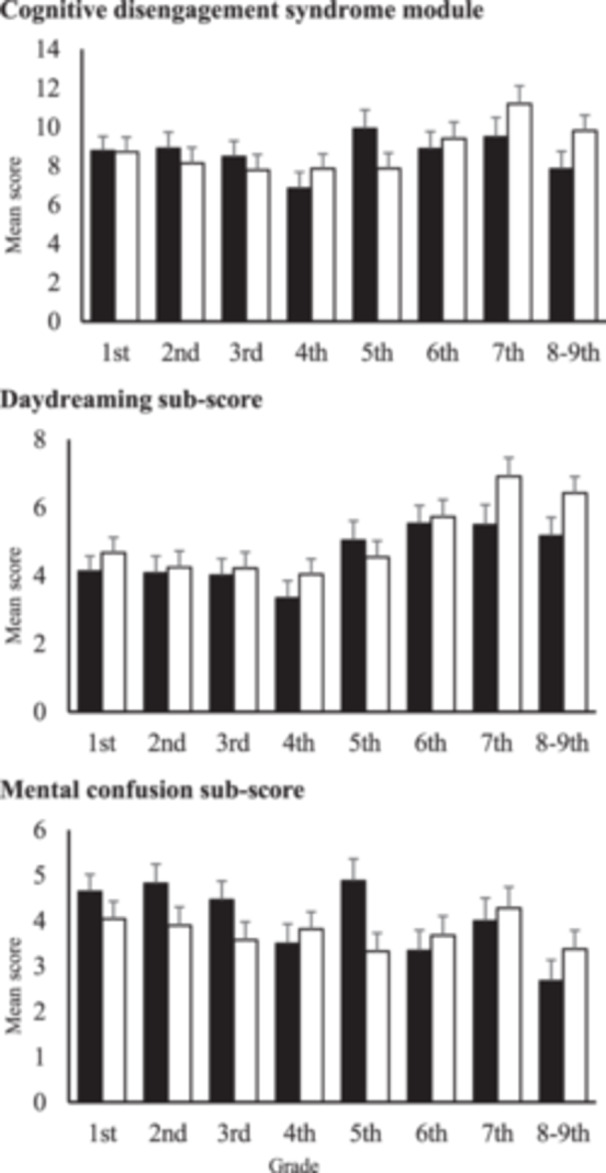
Developmental changes in cognitive disengagement syndrome. Black bars represented boys and white bars represented girls. Error bars represent the standard error of the mean.

We performed two‐way ANOVAs (Sex and Grade) for the CDS module score, as well as daydreaming and mental confusion subscores. No primary effects or interactions found for CDS module score. However, significant primary effects were found in the subscores, but no interactions. For daydreaming, the primary effects of sex and grade were significant (Sex *F* = 3.85, P = 0.05, partial *η* ^2^ = 0.002; Grade *F* = 6.39, P < 0.01, partial *η* ^2^ = 0.019). Girls had higher subscores than boys. The post hoc test for grade revealed that subscore increased with grade level. The 7th‐grade subscore was higher than those of the 1st through 4th grades (P < 0.05). The 8–9th‐grade subscore was higher than those of the 2nd through 4th grades (P < 0.05). In addition, the 6th‐grade subscore was higher than that of the 4th grade (P < 0.05). For mental confusion, there was a main effect of grade only (*F* = 2.38, P = 0.02, partial *η* ^2^ = 0.007). Post hoc tests revealed that as grade increased, the subscore decreased. The 8th‐grade subscore was lower than that of the 1st grade (P < 0.05). No statistically significant differences were observed between the other grades. Developmental changes differed between daydreaming and mental confusion (Figure 2). To provide Japanese norm data and cutoff values, we calculated means and the 85th, 90th, and 95th percentiles for each sex and grade (Table 6).

We examined the comorbidity of CDS and ADHD symptoms using all samples (*n* = 2433) (Table 7). Among the children, 5.7% (*n* = 139) exhibited significant CDS symptoms, and of these, 56.1% (*n* = 78) also had other ADHD symptoms. Inattention was the most common comorbid symptom (ADHD‐IN and CON, *n* = 69). Of the 234 children who exhibited some ADHD symptoms (CDS with ADHD = 78, ADHD only = 156), 33% (*n* = 78) had CDS symptoms.

## DISCUSSION

### Validation

Accurate evaluation of the subjective feelings of others is inherently challenging for observers. Adolescents undergo significant physical, psychological, and social changes and experience frequent emotional fluctuations. Internalizing disorders such as anxiety and depression can be particularly pronounced during this developmental stage. The quality of these assessments is influenced by both the parent's observational proficiency and the degree to which the observed child's emotional states manifest through behaviors such as facial expressions, verbal responses, and daily activities. In this study, the criterion‐related validity in the Anxiety and Depression modules represented the degree of agreement between the parent's observation and the child's subjective judgment. Our results showed that the correlation coefficients were of moderate strength, indicating that parents are also able to assess their children's subjective internalizing disorders.

The criterion‐related validity of the ADHD‐IN, ADHD‐HI, and ODD modules represented the degree of association when the same construct concept was assessed using different questionnaires. Each module was strongly correlated with the results of similar questionnaires traditionally used in Japan, indicating that the same constructs were assessed.

### Concept of CDS

Factor analysis was conducted on the CDS and ADHD items, and CDS items were extracted as factors that were distinct from ADHD. The CDS items were divided into two subfactors: Daydreaming and Mental Confusion. It has been suggested that CDS has potential CDS subdomains such as daydreaming, mental confusion, and hypoactivity. However, there is no consensus on whether these factors are present.^3^ In this study, the items related the hypoactivity (CDS 4 and 9) were included in the Daydreaming factor. It is likely that the two items were challenging to distinguish between hypoactive and daydreaming based on observable behavior for Japanese parents.

Although our results are not based on diagnoses made after clinical interviews, CDS was shown to be highly comorbid with ADHD, particularly the inattention symptom (Table 7). Our findings of comorbidity were consistent with those of a previous study on Spanish children and adolescents.^12^ Additionally, the CDS score was strongly associated with internalizing disorders such as anxiety and depression, whereas it was not associated with externalizing disorders such as ODD (Table 4).

### Reliability

The internal consistency of each module was sufficiently high (*α* > 0.85), except for the Anxiety module (*α* = 0.80). The relatively lower internal consistency of the Anxiety module may be attributed to the items reflecting a range of different anxiety disorders.^13^ The questions in each module can be used to assess common characteristics. As a point to note the ICC, an index of stability, was low in the depression module (ICC 0.57). The reason for the low ICC in this study may be that the survey was conducted before and after a long vacation, which may have altered the children's moods and thus may not reflect the true stability of the module itself. Therefore, the depression module may be sensitive to even small changes in the child, and stability needs to be reexamined during periods when there are fewer changes in the child's environment. Overall, the CABI can be used in Japan to assess internalizing and externalizing disorders.

### Sex and grade differences in CDS

Previous studies^14^, ^15^ have reported that the CDS score is slightly higher in boys, but the effect size is small, or there is no sex difference. Similarly, in the Japanese sample, we found no sex difference when the total score was examined using the CDS as a single factor. When compared separately to the subscores, daydreaming was higher in girls, but the effect size was small. The effect of sex may differ depending on the CDS subfactors. CDS properties undergo at least two distinct developmental changes with increasing daydreaming, whereas mental confusion decreases with the grade. Further studies are needed to determine whether the differences in CDS subdomains are due to biological changes resulting from brain development or psychosocial environmental changes.

### Limitations

This study was conducted during the coronavirus disease 2019 (COVID‐19) outbreak in Japan. Previous research has indicated that COVID‐19 impacts depression levels in children and adolescents.^16^ In our study, the cutoff value for the Depression module may have shifted to a higher threshold compared to the pre‐pandemic period. Therefore, follow‐up studies conducted under conditions unaffected by COVID‐19 are essential to accurately compare the psychological impact of the pandemic.

In this study, traits related to CDS and ADHD were identified using the 95th percentile cutoff value obtained from our sample. However, an ADHD diagnosis necessitates comprehensive evaluations using multiple sources of information to ensure accuracy and reliability. Future research should focus on clinical groups, incorporating methods such as professional interviews, particularly for CDS,^17^ to further enhance the validity and reliability of the findings.

## CONCLUSION

We developed a Japanese version of the CABI and found the following: First, the parents' and children's ratings of internalizing disorders were moderately correlated. The criterion‐related validities of the traditionally used questionnaires were sufficiently high. Second, the CDS has been shown to differ from ADHD symptoms and is significantly related to internalizing problems, even in the Japanese population. The CDS has a two‐factor structure of daydreaming and mental confusion, which are developmentally distinct. Third, normal data were provided so that the CABI could be adapted to the Japanese population.

## AUTHOR CONTRIBUTION

All authors reviewed the results and approved the last version of the manuscript.

*Conceptualization*: Ryusaku Hashimoto, Keitaro Sueda, Yui Tsuji, and Toshinobu Takeda. *Data curation*: Ryusaku Hashimoto, Yui Tsuji, and Yota Nakashima. *Formal analysis*: Ryusaku Hashimoto and Yui Tsuji. *Funding acquisition*: Ryusaku Hashimoto, Keitaro Sueda, Yui Tsuji, Yota Nakashima, Kazuyori Yagyu, and Toshinobu Takeda. *Investigation*: Ryusaku Hashimoto. *Methodology*: Ryusaku Hashimoto, Keitaro Sueda, Yui Tsuji, and Toshinobu Takeda. *Project administration*: Ryusaku Hashimoto. *Resources*: Ryusaku Hashimoto and Kazuyori Yagyu. *Supervision*: Keitaro Sueda, Kazuyori Yagyu, and Toshinobu Takeda. *Visualization*: Ryusaku Hashimoto and Yui Tsuji. *Writing – original draft*: Ryusaku Hashimoto. *Writing – review and editing*: Ryusaku Hashimoto, Keitaro Sueda, Yui Tsuji, Yota Nakashima, Kazuyori Yagyu, and Toshinobu Takeda.

## CONFLICT OF INTEREST STATEMENT

The authors declare no conflicts of interest.

## ETHIC APPROVAL STATEMENT

This study was conducted after review and approval by the Ethics Review Committee of the Health Sciences University of Hokkaido, which conforms to the provisions of the Declaration of Helsinki (Approval No. 20r127120), and after obtaining permission from the City Board of Education.

## PATIENT CONSENT STATEMENT

Informed consent information was provided to all participants on the cover page of the questionnaire. Consent was obtained by completing the questionnaire. All responses were collected anonymously, ensuring that participants' identities remained confidential.

## CLINICAL TRIAL REGISTRATION

This study does not fall under the scope of clinical trial registration requirements.

## Data Availability

The datasets analyzed during this study are available from the corresponding author upon reasonable request.
